# Supplementary material for: Naturally Occurring Chalcones with Aggregation-Induced Emission Enhancement Characteristics
Source: Molecules. 2023 Apr 12;28(8):3412. doi: 10.3390/molecules28083412 (PMC10146426; doi:10.3390/molecules28083412)
Supplement: Supplementary file 1 [file molecules-28-03412-s001.zip › Suplementay materials_chalkony_rev_05.04.pdf]

Naturally occurring Chalcones with Aggregation-Induced Emission Enhancement Characteristics

Suplementarny materials

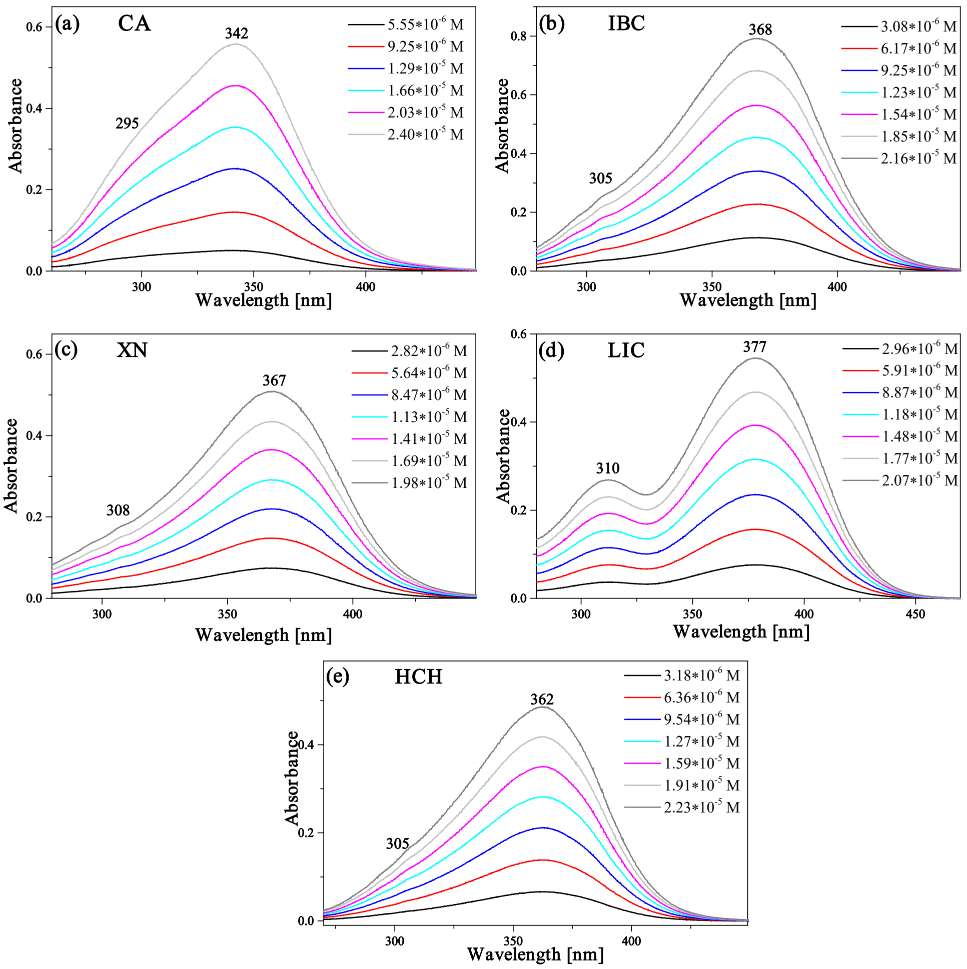

Figure S1. UV-Vis absorption spectra of chalcones CA, IBC, XN, LIC and HCH in methanol solution at different concentration of compounds.

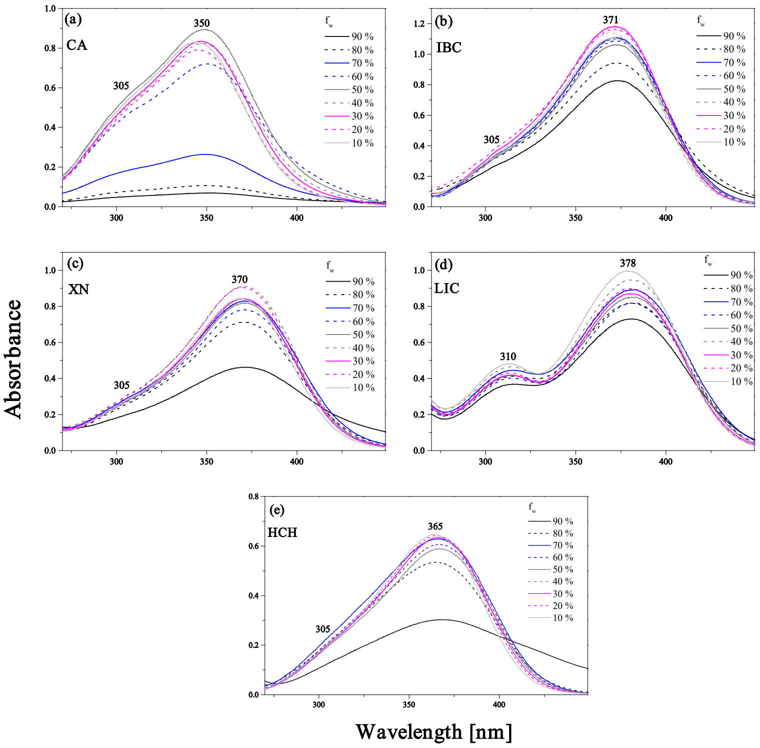

Figure S2. UV-Vis absorption spectra of chalcones CA, IBC, XN, LIC and HCH in various MeOH:H_2O proportion.

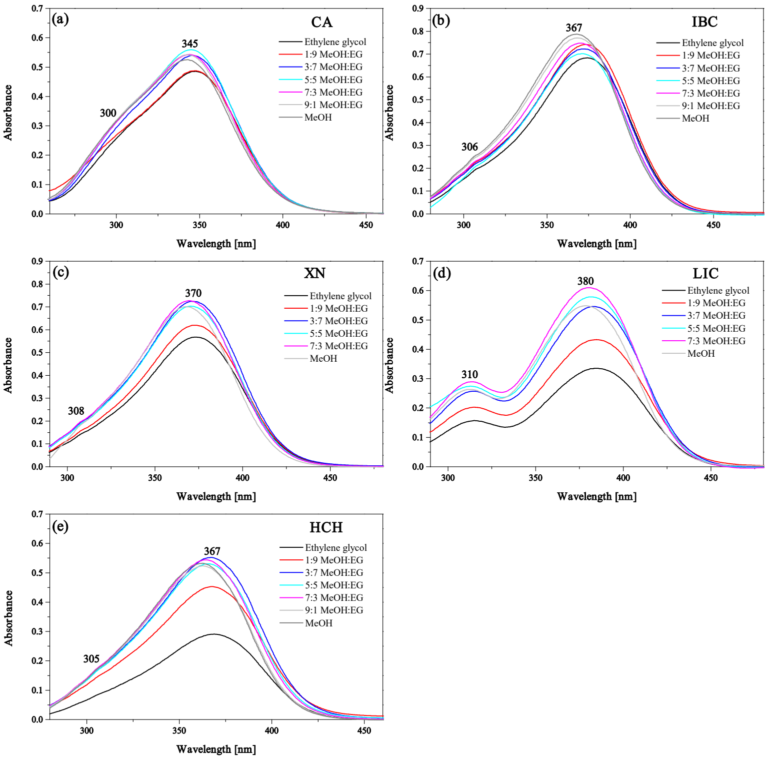

Figure S3. UV-Vis absorption spectra of chalcones CA, IBC, XN, LIC and HCH in MeOH:ethylene glycol (EG) solution.

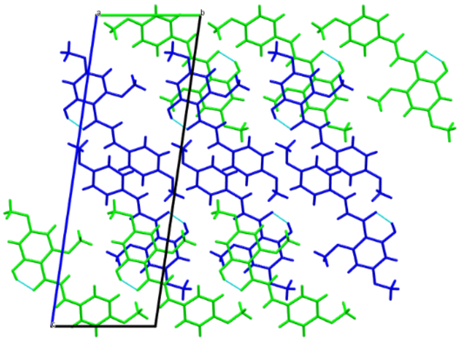

(a) HCH view along [100]

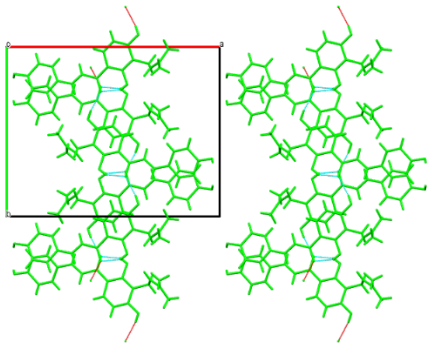

(b) IBC view along [001]

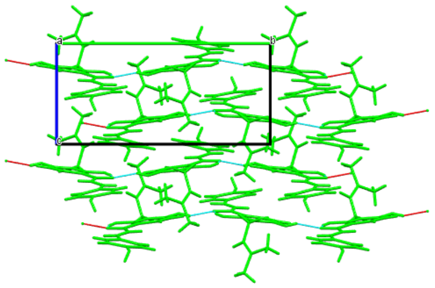

(c) IBC view along [100]

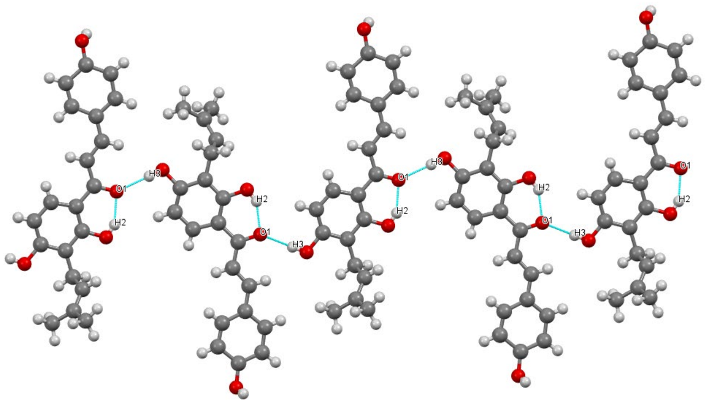

(d) IBC motif of infinite chains C(8)

Figure S4. The HCH and IBC crystal lattice. A packing diagram of the HCH chain interactions within the layers in HCH crystal (panel a) and IBC crystal (panel b and c). A layered section of the IBC structure to show the homomolecular motif of infinite chains C(8) observed in the layers of IBC molecules (panel d).

Table S1. Spectroscopic data for CA, IBC, XN, LIC and HCH dissolved in different solvents: maximum absorbance, fluorescence and stokes shift.

Compound	Solvent	Absorbance [nm]	Absorbance [cm^{-1}]	Fluorescence [nm]	Fluorescence [cm^{-1}]	Stokes shift [nm]	Stokes shift [cm^{-1}]
**LIC**	MeOH	380	26315.79	490	20408.16	110	5907.63
	H_2O	380	26315.79	514	19455.25	134	6860.54
	EG	384	26041.67	500	20000.00	116	6041.67
	Chloroform	360	27777.78	446	22421.52	86	5356.25
	EPOXY resin	375	26666.67	513	19493.18	138	7173,49
**CA**	MeOH	342	29239.77	443	22573.36	101	6666.40
	H_2O	357	28011.20	440	22727.27	83	5283.93
	EG	348	28735.63	430	23255.81	82	5479.82
	Chloroform	343	29154.52	400	25000.00	57	4154.52
	EPOXY resin	365	27397.26	475	21052.63	110	6344,63
**HCH**	MeOH	363	27548.21	408	24509.80	45	3038.41
	H_2O	370	27027.03	410	24390.24	40	2636.78
	EG	368	27173.91	423	23640.66	55	3533.25
	EPOXY resin	365	27397.26	451	22172.95	86	5224,31
	Chloroform	365	27397.26	415	24096.39	50	3300.87
**XN**	MeOH	368	27173.91	412	24271.84	44	2902.07
	H_2O	368	27173.91	413	24213.08	45	2960.83
	EG	373	26809.65	424	23584.91	51	3224.75
	Chloroform	366	27322.40	410	24390.24	44	2932.16
	EPOXY resin	380	26315.79	464	21551.72	84	4764,07
**IBC**	MeOH	367	27247.96	415	24096.39	48	3151.57
	H_2O	371	26954.18	426	23474.18	55	3480.00
	EG	373	26809.65	425	23529.41	52	3280.24
	Chloroform	367	27247.96	413	24213.08	46	3034.88
	EPOXY resin	380	26315.79	536	18656.72	156	7659,07

Table S2. Crystallographic parameters and refinement details for the HCH, CA and IBC crystals.

**Compound**	**HCH**	**CA**	**IBC**
Empirical formula	$C_{18}H_{18}O_5$	$C_{16}H_{14}O_4$	$C_{20}H_{20}O_4$
Temperature (K)	293(2)	293(2)	296(2)
Crystal system	Triclinic	Monoclinic	Monoclinic
Space group	P-1	$P2_1/c$	$P2_1/c$
a (Å)	7.4636(5)	25.7114(6)	19.354(4)
b (Å)	8.4407(3)	15.3506(3)	15.2873(18)
c (Å)	25.5378(12)	6.8717(2)	7.2681(11)
α (°)	97.847(3)	90	90
β (°)	93.939(6)	97.058(3)	97.215(18)
γ (°)	95.084(4)	90	90
V (Å^3)	1582.17(15)	2691.60(12)	2133.4(6)
Z	4	8	4
Calculated density (g·cm^{-3})	1.320	1.3339	1.010
Absorption coeffcient (mm^{-1})	0.796	0.792	0.568
F(000)	666.4	1140	688.0
θ range for data collection (°)	10.52 – 141.66	4.499 – 68.185	7.392 – 137.066
Index ranges	$-8 \leq h \leq 8$	$-26 \leq h \leq 30$	$-22 \leq h \leq 23$
	$-8 \leq k \leq 9$	$-17 \leq k \leq 18$	$-18 \leq k \leq 17$
	$-30 \leq l \leq 30$	$-7 \leq l \leq 8$	$-8 \leq l \leq 8$
Reflections collected	20179/5705	25350/4917	15785/3809
	(R_{int}=0.0575)	(R_{int} = 0.0355)	(R_{int} = 0.1271)
Data/restraints/parameter	5705/0/423	2073/0/368	3809/0/223
Goodness-of-fit on F^2	1.036	1.0594	1.918
Final R indices R [I>2σ(I)]	R1 = 0.0788	R1 = 0.0678	R1 = 0.2758
	wR2 = 0.2327	wR2 = 0.2274	wR2 = 0.5307
Final R indices (all data)	R1 = 0.0953	R1 = 0.0839	R1 = 0.3082
	wR2 = 0.2634	wR2 = 0.2409	wR2 = 0.5557
Largest diff. peak and hole/ eÅ^{-3}	0.32/-0.27	0.3/−0.3	1.06/−0.73
**CCDC Number**	**2239026**	**2014912**	**2243539**

Table S3. Bond length for HCH and IBC crystals.

**HCH crystal**					
**Atom**	**Atom**	**Length [Å]**	**Atom**	**Atom**	**Length [Å]**
O3A	C4A	1.352(3)	O4B	C6B	1.363(2)
O3A	C16A	1.435(3)	O3B	C4B	1.356(3)
O4A	C6A	1.360(2	O4B	C17B	1.424(3)
O4A	C17A	1.424(3	O3B	C16B	1.420(3)
O2A	C2A	1.345(3)	O2B	C2B	1.340(3)
O5A	C13A	1.367(3)	O5B	C13B	1.364(3)
O5A	C18A	1.424(4)	O5B	C18B	1.423(4)
O1A	C7A	1.263(3)	O1B	C7B	1.256(3)
C1A	C6A	1.422(3)	C3B	C4B	1.368(3)
C1A	C2A	1.424(3)	C3B	C2B	1.389(3)
C1A	C7A	1.459(3)	C1B	C6B	1.422(3)
C4A	C3A	1.369(3)	C1B	C2B	1.422(3)
C4A	C5A	1.402(3)	C1B	C7B	1.465(3)

C3A	C2A	1.387(3)	C8B	C9B	1.323(3)
C6A	C5A	1.370(3)	C8B	C7B	1.465(3)
C10A	C9A	1.446(3)	C6B	C5B	1.371(3)
C10A	C15A	1.397(4)	C5B	C4B	1.394(3)
C10A	C11A	1.385(3)	C10B	C15B	1.401(3)
C8A	C9A	1.332(3)	C10B	C9B	1.463(3)
C8A	C9A	1.332(3)	C10B	C11B	1.381(3)
C15A	C14A	1.363(4)	C12B	C13B	1.386(3)
C13A	C12A	1.366(4)	C12B	C11B	1.375(3)
C13A	C14A	1.389(4)	C12B	C11B	1.375(3)
C11A	C12A	1.380(4)	C14B	C13B	1.377(4)
**IBC crystal**					
**Atom**	**Atom**	**Length [Å]**	**Atom**	**Atom**	**Length [Å]**
O2	C16	1.370(8)	C10	C9	1.444(13)
O3	C14	1.371(10)	C5	C4	1.428(13)
O1	C10	1.262(9)	C5	C6	1.412(13)
C11	C16	1.387(12)	C18	C17	1.472(11)
C11	C10	1.477(11)	C18	C19	1.386(12)
C11	C12	1.426(9)	C19	C21	1.484(13)
C13	C14	1.366(11)	C19	C20	1.434(14)
C13	C12	1.348(11)	C4	C3	1.302(15)
C16	C15	1.393(11)	C6	C7	1.370(15)
C14	C15	1.409(10)	O4	C2	1.314(14)
C8	C9	1.304(12)	C7	C2	1.416(19)
C8	C5	1.396(12)	C2	C3	1.484(19)
C15	C17	1.507(12)			
